# Effects of Luteolin on Human Breast Cancer Using Gene Expression Array: Inferring Novel Genes

**DOI:** 10.3390/cimb44050142

**Published:** 2022-05-09

**Authors:** Shih-Ho Wang, Chin-Hu Wu, Chin-Chuan Tsai, Tai-Yu Chen, Kuen-Jang Tsai, Chao-Ming Hung, Chia-Yi Hsu, Chia-Wei Wu, Tsung-Hua Hsieh

**Affiliations:** 1 Department of Surgery, Kaohsiung Chang Gung Memorial Hospital, Chang Gung University College of Medicine, Kaohsiung 83301, Taiwan; ndmcm81@yahoo.com.tw; 2 Division of General Surgery, Kaohsiung Chang Gung Memorial Hospital, Chang Gung University College of Medicine, Kaohsiung 83301, Taiwan; 3 Department of Obstetrics and Gynecology, Kaohsiung Medical University Hospital, Kaohsiung Medical University, Kaohsiung 80708, Taiwan; 810080@kmuh.org.tw (C.-H.W.); husonweihsu@hotmail.com (C.-Y.H.); 4 Department of Chinese Medicine, E-Da Hospital, Kaohsiung 82445, Taiwan; ed103622@edah.org.tw (C.-C.T.); ed107076@edah.org.tw (T.-Y.C.); 5 Department of Surgery, E-Da Cancer Hospital, I-Shou University, Kaohsiung 82445, Taiwan; ed108937@edah.org.tw (K.-J.T.); ed100647@edah.org.tw (C.-M.H.); 6 Department of Medical Research, E-Da Hospital/E-Da Cancer Hospital, I-Shou University, Kaohsiung 82445, Taiwan; snoopy79101@gmail.com

**Keywords:** luteolin, breast cancer and gene expression array

## Abstract

*Taraxacum officinale* (dandelion) is often used in traditional Chinese medicine for the treatment of cancer; however, the downstream regulatory genes and signaling pathways mediating its effects on breast cancer remain unclear. The present study aimed to explore the effects of luteolin, the main biologically active compound of *T. officinale*, on gene expression profiles in MDA-MB-231 and MCF-7 breast cancer cells. The results revealed that luteolin effectively inhibited the proliferation and motility of the MDA-MB-231 and MCF-7 cells. The mRNA expression profiles were determined using gene expression array analysis and analyzed using a bioinformatics approach. A total of 41 differentially expressed genes (DEGs) were found in the luteolin-treated MDA-MB-231 and MCF-7 cells. A Gene Ontology analysis revealed that the DEGs, including AP2B1, APP, GPNMB and DLST, mainly functioned as oncogenes. The human protein atlas database also found that AP2B1, APP, GPNMB and DLST were highly expressed in breast cancer and that AP2B1 (cut-off value, 75%) was significantly associated with survival rate (*p* = 0.044). In addition, a Kyoto Encyclopedia of Genes and Genomes pathway analysis revealed that the DEGs were involved in T-cell leukemia virus 1 infection and differentiation. On the whole, the findings of the present study provide a scientific basis that may be used to evaluate the potential benefits of luteolin in human breast cancer. Further studies are required, however, to fully elucidate the role of the related molecular pathways.

## 1. Introduction

Breast cancer is the most common type of cancer among women worldwide; ~2.1 million women are affected by breast cancer each year. In addition, breast cancer has the highest mortality rate among all cancers. In 2018, an estimated 600,000 women worldwide succumbed to this disease, accounting for ~15% of all cancer-related deaths among women. According to the statistics, although the incidence of breast cancer in women in more developed regions is higher, the incidence of breast cancer is increasing in almost all regions worldwide [1,2]. Breast cancer often metastasizes to distant organs, such as the bones, liver, lungs and brain, which is the primary cause of treatment failure. Currently, mammography is the most basic screening method for detecting breast cancer; mammography can detect breast cancer at an early stage and can thus effectively reduce the mortality rate [3,4]. According to a previous statistical analysis, breast cancer is currently the leading cause of cancer-related mortality among women in Taiwan and the fourth leading cause of cancer-related mortality among women worldwide; the number of deaths is also increasing annually [5]. The early diagnosis of the disease can result in a good prognosis and a high survival rate. Thus, screening is crucial for early detection [6,7].

Luteolin(3′,4′,5′,7′-tetrahydroxyflavonoid) is the main component of the leaves of *Taraxacum officinale* [8]. *Taraxacum officinale*, commonly known as dandelion, is a perennial herb belonging to the *Asteraceae* family [9]. It is native to Eurasia but has been found worldwide. It has been used as a traditional and modern herbal system in several countries. These ingredients are non-toxic and can be used as anti-inflammatory drugs; diuretics; digestive stimulants; insulin stimulants; and antitumor, antidiabetic and antioxidant agents as well as to prevent liver and testicular damage [10,11,12,13,14]. Luteolin is a common flavonoid [15,16] that can be found in a number of plants, fruits, vegetables and herbs [17]. It was previously found that luteolin can increase the effectiveness of paclitaxel to induce breast cancer cell apoptosis by inhibiting STAT3 [18]. In addition, *Taraxacum officinale* leaves include caffeic and chlorogenic acid [8]. Caffeic acid inhibits cell proliferation by mediating estrogen receptor levels in human breast cancer cells [19]. Chlorogenic acid induces apoptosis by regulating NANOG, POU5F1 and SOX2 in human lung cancer cells [20].

Although several studies have investigated the mechanisms and cellular responses of luteolin in cancer, these mechanisms are complex and have not been fully elucidated. Therefore, the present study performed a comprehensive analysis of various genetic expression modifications under treatment with luteolin using full gene array and bioinformatic analyses.

## 2. Materials and Methods

### 2.1. Cell Culture and Reagent

The human breast cancer cell lines MDA-MB-231 and MCF-7 were obtained from the American Type Culture Collection (ATCC). The cells were cultured in DMEM supplemented with 10% FBS and 1% penicillin/streptomycin and incubated at 37 °C in 5% CO_2_. Luteolin, caffeic acid and chlorogenic acid were purchased from Sigma-Aldrich (St. Louis, MO, USA).

### 2.2. Cell Viability Assay

The cell viability assay was performed using a Cell Counting Kit-8 (CCK-8) according to the manufacturer’s protocol. Briefly, 1 × 10^3^ MCF-7 and MDA-MB-231 cells were plated in 96-well plates and incubated with luteolin at various concentrations (0, 10, 20, 40 and 80 μM) and for different periods of time (24, 48 and 72 h). After specifying the conditions, the medium was removed, and the cells were incubated with CCK-8 reagent diluted in culture medium at 37 °C for 2 h. The absorbance was analyzed at 450 nm using a microplate reader.

### 2.3. Wound Healing Assay

Breast cancer cells (5 × 10^5^) were seeded in six-well plates. Following 24 h of incubation, scratches were created in each well using a sterile yellow pipette tip, and images of the edge of the scratch to the wounded area in the wells were acquired using an inverted microscope at 0 and 48 h.

### 2.4. RNA Extraction and Reverse Transcription-Quantitative

*RT-qPCR*. Total RNA was extracted from the breast cancer cells using TRI^®^ Reagent RNA Isolation Reagent (Sigma-Aldrich; Merck KGaA), and cDNA was synthesized using a cDNA Synthesis kit according to the manufacturer’s instructions. cDNA was PCR amplified with the following primers: adaptor-related protein complex 2 subunit beta 1 (AP2B1) forward, 5′-GGCTTTGGGAGACAAGTTGA-3′, and reverse, 5′-CAGCAGTGGCTGGTCTGATA-3′; amyloid beta precursor protein (APP) forward, 5′-CCTTACAGTGGAGGCTTGTTAGA-3′, and reverse, 5′ GTATGTGACCTAACAGGAGCATC-3′; glycoprotein Nmb (GPNMB) forward, 5′-GAATTTCGGAGGGACACAAA-3′, and reverse, 5′-GGGGTAACTGGTGGTCAATG-3′; dihydrolipoamide S-succinyltransferase (DLST) forward, 5′-GGCATACCATGGGTTGAATC-3′, and reverse, 5′-TTCTCAGGGGCTATGCACTT-3′; and GAPDH forward, 5′-CATCAAGAAGGTGGTGAAGCAG-3′, and reverse, 5′-GTGTCGCTGTTGAAGTCAGAG-3′. Relative quantification was performed by normalizing the target mRNA expression levels to the levels of GAPDH and was calculated using the 2^−ΔΔCq^ method.

### 2.5. Array Hybridization and Data Analysis

Gene expression profiles were analyzed using an Agilent Technologies array SurePrint G3 Human Gene Expression 8 × 60 K microarray according to the manufacturer’s instructions. For transcription, Cy3-labeled RNA was amplified with a Low Input Quick-Amp Labeling kit (Agilent Technologies, Inc., Santa Clara, CA, USA). Fragmented, labeled cRNA was hybridized to an Agilent SurePrint Microarray, and scanned images were analyzed using Feature Extraction 10.7.3.1 software (Agilent Technologies, Inc.). Differential gene expression was normalized by quantile normalization of the raw signal data. The clusterProfiler package was used for Gene Ontology (GO) and Kyoto Encyclopedia of Genes and Genomes (KEGG) analyses by Welgen, Inc. (Worcester, MA, USA) A flowchart illustrating the gene expression profiling process is shown that total RNA was extracted from the cells and sent for whole-genome microarray analysis followed by bioinformatics analyses.

### 2.6. Statistical Analysis

The data are the mean ± SD from three independent experiments. * *p* < 0.05 vs. untreated control (the data were analyzed using a two-tailed Student’s *t*-test). In addition, data from the human protein atlas database were analyzed by descriptive statistics programs in GraphPad.

## 3. Results

### 3.1. Luteolin Inhibits the Viability and Wound Healing Ability of Human Breast Cancer Cells

**Figure 1:**
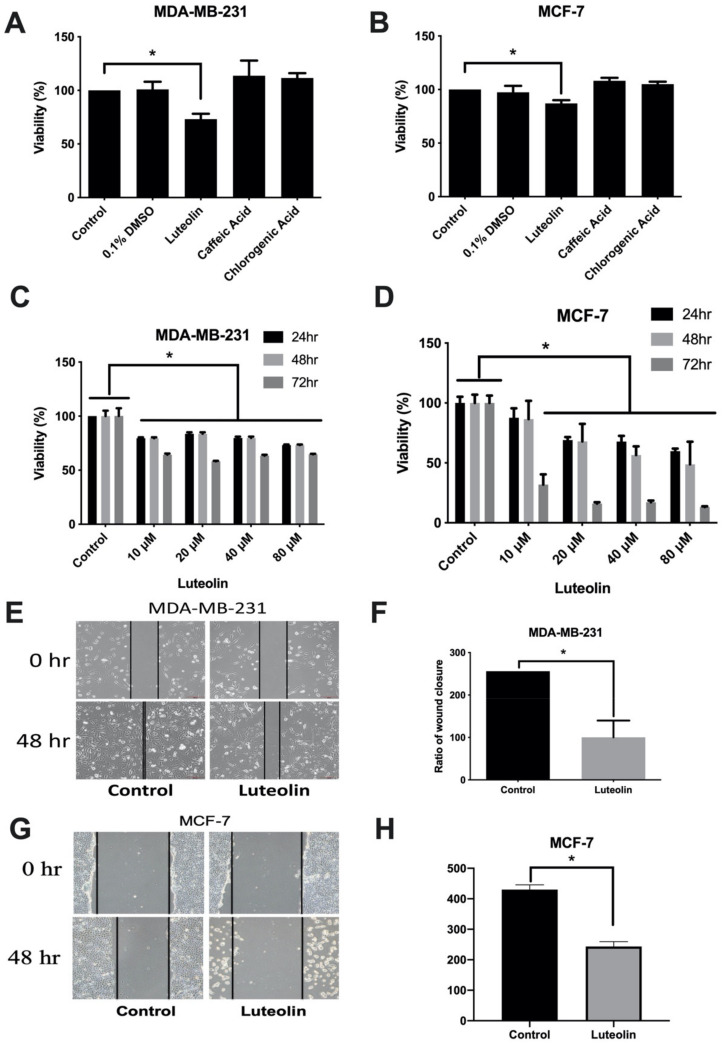
Luteolin mediates the proliferation and migration of breast cancer cells. Breast cancer cells were exposed to 20 µM luteolin, caffeic acid and chlorogenic acid for 24 h. The growth of (**A**) MDA-MB-231 and (**B**) MCF-7 cells was analyzed using a CCK-8 assay. The dose-/time-dependent effects of luteolin were also analyzed using a CCK-8 assay in (**C**) MDA-MB-231 and (**D**) MCF-7 cells. (**E**,**G**) Migration was assessed using a wound healing assay. The horizontal dashed line indicates the width of the wound. (**F**,**H**) Results of quantitative analysis of the wound healing assay. The data are presented as the mean ± SD of three experiments. * *p* < 0.05 versus untreated control.

To examine the effects of luteolin on breast cancer, three main ingredients of *Taraxacum officinale* leaves including luteolin, caffeic and chlorogenic acid were used and cell viability was examined using CCK-8 assays. The results revealed that only luteolin significantly inhibited the viability of the MDA-MB-231 estrogen receptor (ER)^-^/progesterone receptor (PR)^-^/human epidermal growth factor receptor (HER^-^) and MCF-7 (ER^+^/PR^+^) cells (Figure 1A,B). The concentration- and time-dependent effects of luteolin on the viability of MCF-7 and MDA-MB-231 cells were further analyzed. Similarly, it was found that luteolin more effectively inhibited cell viability in a time- and concentration-dependent manner (Figure 1C,D). In addition, the effects of luteolin on cell motility were analyzed using a wound healing assay. The results revealed that luteolin inhibited cell motility and migration (Figure 1E–H). These results suggested that luteolin can effectively inhibit breast cancer cell proliferation and motility.

### 3.2. Identification of Potential Gene Expression Alterations in Luteolin-Treated Breast Cancer Cells

**Figure 2:**
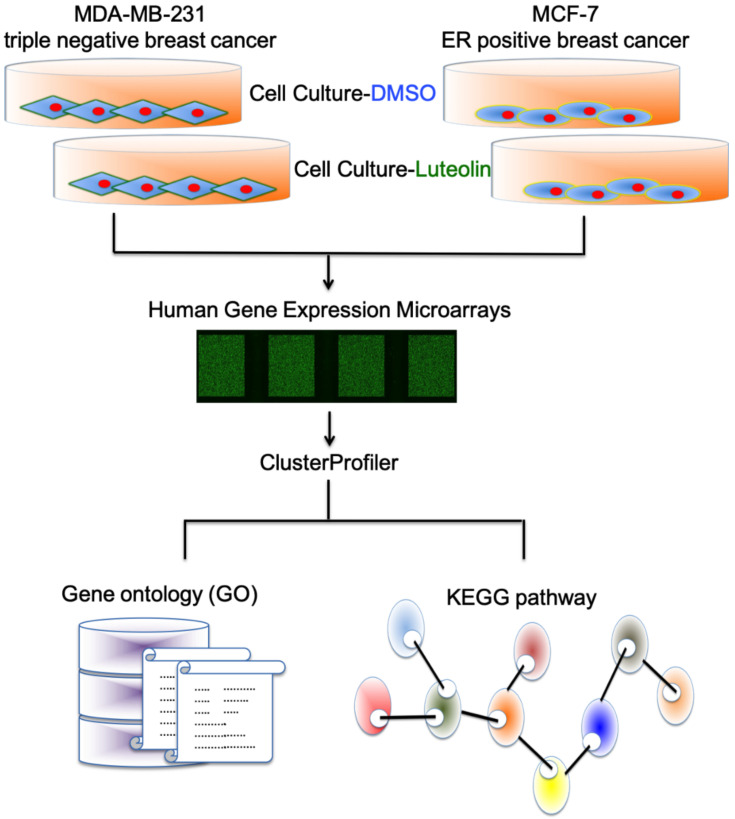
Investigation of luteolin-related changes in MDA-MB-231 and MCF-7 cells. Flowchart illustrating the methods for investigating various gene expression changes in luteolin-treated MDA-MB-231 and MCF-7 cells by gene expression array and bioinformatic approaches.

In the past, the majority of studies on luteolin in breast cancer focused on triple-negative breast cancer. Therefore, in the present study, a whole-genome microarray was used to analyze the genes regulated by luteolin in the MCF-7 and MDA-MB-231 cells (Figure 2).

**Figure 3:**
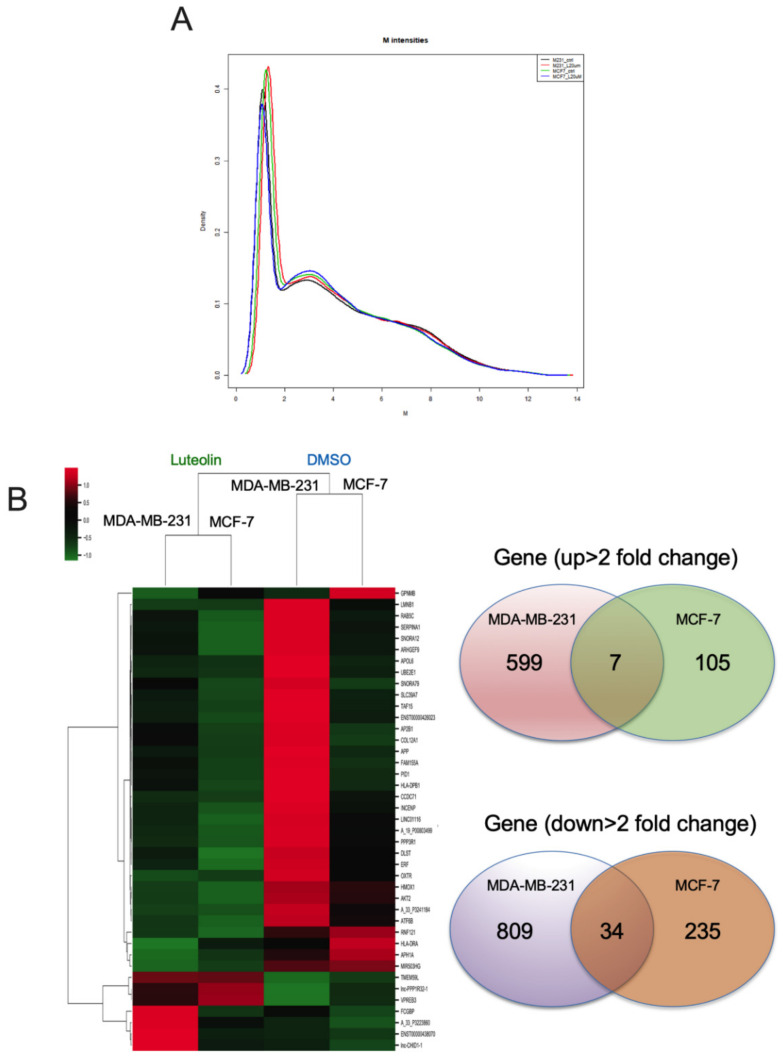
Fold changes in mRNA expression in luteolin-treated breast cancer cells. (**A**) Signal distribution across the ChIP channel in the density plots. (**B**) Heatmap visualization of the gene microarray analysis results illustrating the differentially expressed genes (total 1789 genes) with fold changes > ±2.0. Venn diagram of genes illustrating that luteolin mediated 711 genes that were upregulated and 1078 genes that were downregulated in breast cancer.

The density plots indicate the signal distribution across a chip (Figure 3A). A heatmap plot of differentially upregulated (top panel) and downregulated (bottom panel) genes in the luteolin-treated MDA-MB-231 and MCF-7 breast cancer cell lines was created. Genes with >±2-fold changes in expression and a −log10 (*p*-value) (Figure 3B) were selected for further analyses. Microarray analysis revealed that the expression of 7 upregulated and 34 downregulated genes was changed by >2-fold in the MCF-7/MDA-MB-231 cells treated with luteolin compared with the control cells. These 41 differentially regulated genes are listed in Table 1.

### 3.3. Validation of Differentially Expressed Genes (DEGs) in Response to Luteolin

**Figure 4:**
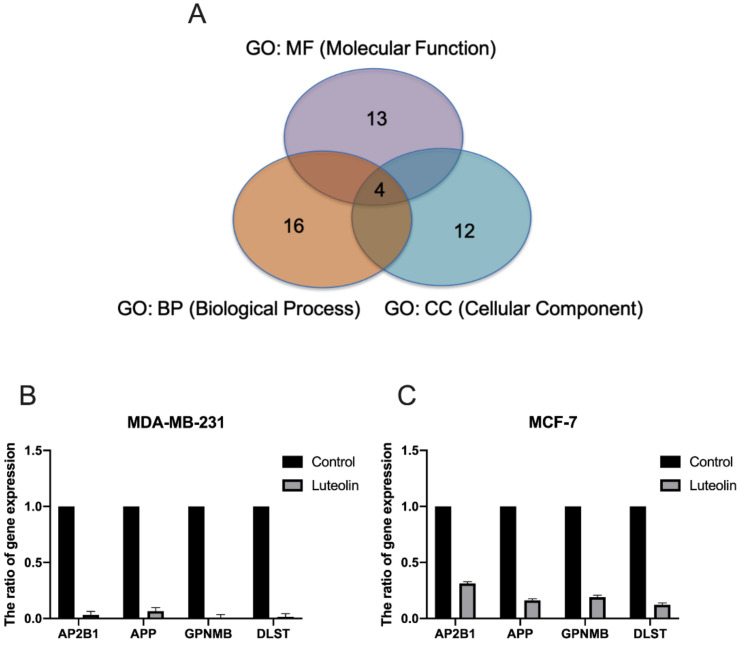
Effect of luteolin on the expression of selected genes in breast cancer cells. (**A**) Four identified genes, AP2B1, APP, GPNMB and DLST, were found to be involved in ‘molecular function’, ‘biological processes’ and ‘cellular components’. (**B**,**C**)The expression of AP2B1, APP, GPNMB and DLST was examined in MCF-7 and MDA-MB-231 cells treated with 20 µM luteolin using reverse transcription-quantitative PCR. The data are presented as the mean ± SD of three experiments. AP2B1, adaptor related protein complex 2 subunit beta 1; APP, amyloid beta precursor protein; GPNMB, glycoprotein Nmb; DLST, dihydrolipoamide S-succinyltransferase.

Subsequently, network analyses of the 41 genes with a differential expression mediated by luteolin were performed using ingenuity pathway analysis (IPA) software. The biological processes and molecular function terms, i.e., ‘molecular function (MF)’, ‘biological process (BP)’ and ‘cellular component (CC)’, associated with the DEGs in the luteolin-treated breast cancer cells are listed in Figure 4 and Table 2. The biological processes and molecular functions associated with DEGs in the luteolin-treated breast cancer cells are listed in Table 2. In the GeneGo Cellular Process network, a total of 13 genes (AP2B1, HLA-DRA, PPP3R1, HLA-DPB1, OXTR, SERPINA1, APP, ATF6B, FAM155A, LMNB1, GPNMB, SLC39A7 and DLST) were identified in the MF category, 16 genes (HMOX1, AP2B1, AKT2, APP, GPNMB, OXTR, PID1, PPP3R1, SLC39A7, APH1A, ATF6B, RNF121, DLST, COL12A1, UBE2E1 and GPNMB) were identified in the BP category, and 12 genes (SERPINA1, HLA-DRA, HLA-DPB1, AP2B1, APH1A, RAB5C, AKT2, APP, GPNMB, LMNB1, DLST and INCENP) were identified in the CC category.

Further assessment of the microarray analysis results revealed that the AP2B1, APP, GPNMB and DLST genes were consistently identified in the MF, BP and CC categories (Figure 4A). To confirm the reliability of the fold changes identified with microarray analysis, the AP2B1, APP, GPNMB and DLST genes were analyzed using RT-qPCR. The results revealed that all the examined genes were downregulated by luteolin treatment in the MDA-MB-231 and MCF-7 human breast cancer cell lines (Figure 4B).

### 3.4. External Validation of AP2B1, APP, GPNMB and DLST in Cell Viability, Human Protein Atlas (HPA) Databases and KEGG Pathways

**Figure 5:**
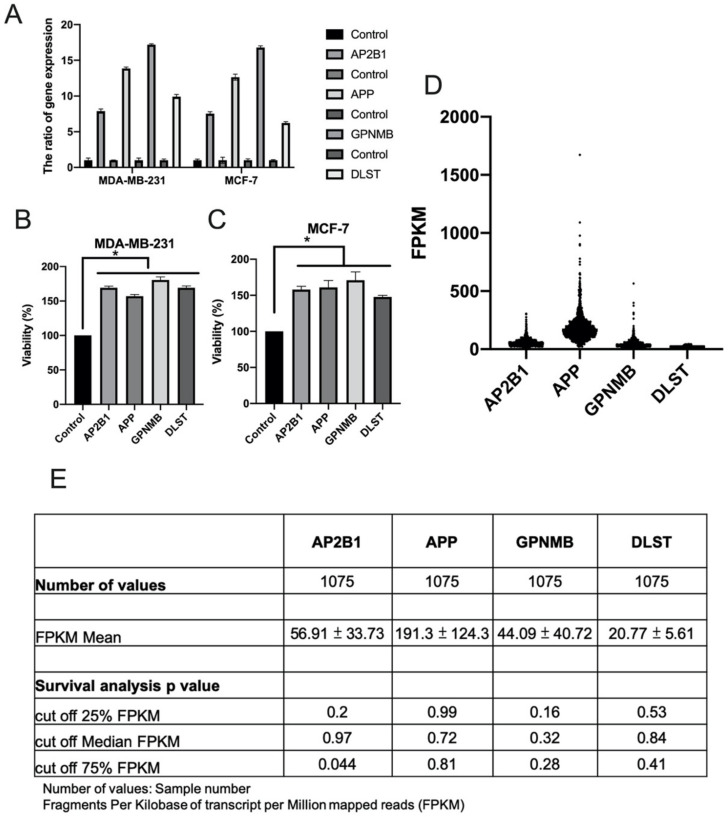
Role of AP2B1, APP, GPNMB and DLST in cell viability and survival. A total of 1 μg target gene plasmid was used for the transfection of MDA-MB-231 and MCF-7 breast cancer cell lines. (**A**) Gene expression was analyzed using reverse transcription-quantitative PCR, and (**B**,**C**) cell proliferation was analyzed using CCK-8 assay. Data are the mean ± SD from three independent experiments. * *p* < 0.05 vs. untreated control (data were analyzed using a two-tailed Student’s *t*-test). (**D**,**E**) Gene expression and survival rate were analyzed by fragments per kilobase per million of AP2B1, APP, GPNMB and DLST through the human protein atlas database. AP2B1, adaptor-related protein complex 2 subunit beta 1; APP, amyloid beta precursor protein; GPNMB, glycoprotein Nmb; DLST, dihydrolipoamide S-succinyltransferase.

To further verify the effects of four genes AP2B1, APP, GPNMB and DLST on breast cancer, plasmids of the four genes were transfected into breast cancer cell lines and gene overexpression was analyzed using RT-qPCR (Figure 5A). Cell viability assay revealed that AP2B1, APP, GPNMB and DLST increased the growth of the MDA-MB-231 (Figure 5B) and MCF-7 cells (Figure 5C). In addition, the HPA database demonstrated [21] that AP2B1, APP, GPNMB and DLST were highly expressed in breast cancer (Figure 5D); the increased expression of AP2B1 (cut-off value, 75%) was significantly associated with the survival rate (*p* = 0.044) of 1075 patients with breast cancer (Figure 5E).

**Figure 6:**
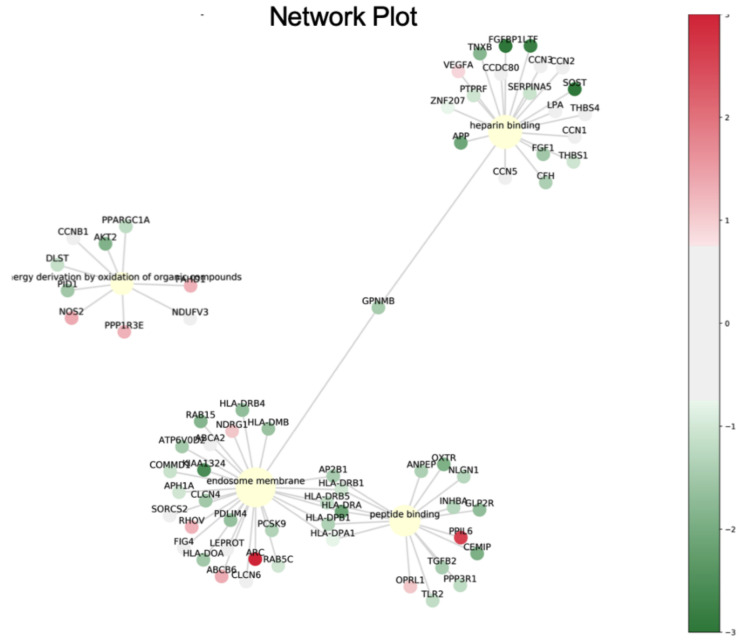
Pathway network of the proteins differentially expressed in response to luteolin treatment.

The data suggested that the four genes, AP2B1, APP, GPNMB and DLST, were highly expressed in breast cancer and that AP2B1 was a suitable prognostic marker for breast cancer. The pathway network of these candidate genes mediating the response to luteolin is illustrated schematically in Figure 6.

**Figure 7:**
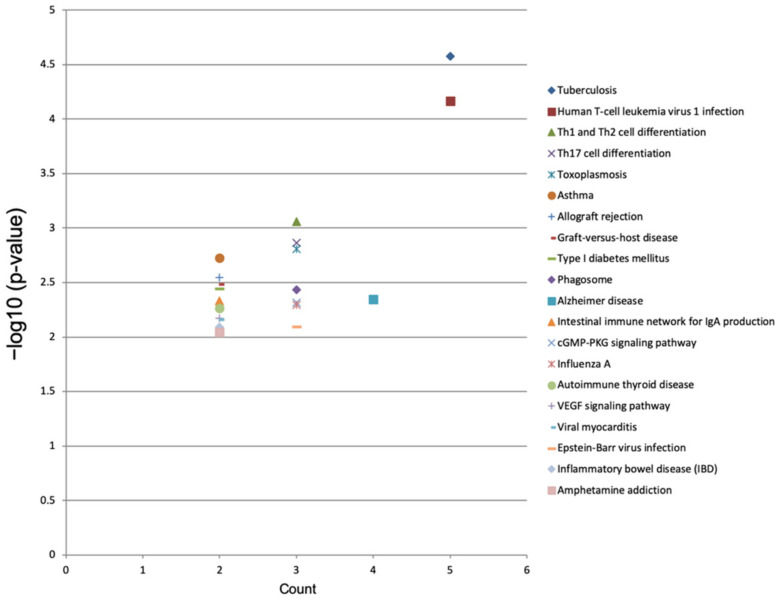
KEGG pathway enrichment analysis of differentially expressed genes in luteolin-treated cells. In the image, the top 20 KEGG pathways with −log10 (*p* value) and multiple enrichment are plotted. A higher fold enrichment value and −log10 (*p* value) indicate that the expression level of the pathway is higher and more reliable.

The top 20 KEGG pathways enriched in the dysregulated genes were identified using mRNA sequencing data. The tuberculosis (*p* = 0.00003), human T-cell leukemia virus 1 infection (*p* = 0.00007), Th1 and Th2 cell differentiation (*p* = 0.0009), Th17 cell differentiation (*p* = 0.0014), toxoplasmosis (*p* = 0.0016), asthma (*p* = 0.0019), allograft rejection (*p* = 0.0028), graft-versus-host disease (*p* = 0.0033), type I diabetes mellitus (*p* = 0.0036), phagosome (*p* = 0.0037), Alzheimer’s disease (*p* = 0.0045), intestinal immune network for IgA production (*p* = 0.0047) and cGMP-PKG signaling (*p* = 0.0048) pathways were significantly enriched in the dysregulated genes of the luteolin-treated breast cancer cells (Figure 7 and Table 3).

## 4. Discussion

Natural herbs and plants continue to play an important role in drug research and development, particularly in cancer research. Over the past 20 years, 50% of all anticancer drugs approved worldwide are natural products or components of natural products [22]. The present study found that luteolin inhibited breast cancer cell proliferation and invasion and comprehensively surveyed the differential expression of mRNAs in mediating the effects of luteolin on MDA-MB-231 and MCF-7 cells using microarray and bioinformatics analyses. Previous studies have found that luteolin inhibits inflammation, allergies and cancer through its antioxidant and pro-oxidative abilities. It has been used in traditional Chinese medicine to prevent and treat various diseases including hypertension, inflammatory diseases, Alzheimer’s disease [23] and many types of cancer [17]. Recent studies have also found that luteolin significantly inhibits cancer cell proliferation and metastasis through the AKT/mTOR/MMP9 signaling pathway in androgen receptor-positive triple-negative breast cancer. It has also been demonstrated that luteolin inhibits the in vitro and in vivo proliferation of lung cancer cells by targeting LIMK1 [24]. The present study identified four genes (AP2B1, APP, GPNMB and DLST) that were significantly downregulated by luteolin in breast cancer cell lines. These genes were associated with drug resistance, cell proliferation, macrophages, apoptosis and HDAC inhibition.

AP2B1 is a subunit of adaptor-related protein complex 2, a clathrin assembly protein complex, and is involved in clathrin-mediated endocytosis. The resistance phenotype to cancer drugs is associated with an increase in the protein level of AP2B1 in non-small cell lung cancer [25]. A previous study found that luteolin induced apoptosis by blocking the PI3K/AKT/mTOR pathway in tamoxifen-resistant breast cancer cells [26]. This result led to the hypothesis that luteolin can influence drug resistance by regulating AP2B1. APP is an evolutionarily conserved protein. APP is overexpressed in several types of cancer, including breast, colon, lung and pancreatic cancer, and promotes cell proliferation, invasion and migration. In breast cancer, APP has been shown to mediate cell proliferation and motility through the AKT signaling pathway [27,28]. In addition, APP has been shown to regulate proliferation through the ERK signaling pathway in colon cancer [29,30]. APP also increases proliferation and leads to cell size abnormalities in both lung and pancreatic cancer [31,32,33]. The same results were also found in the present study; in addition, it was found that luteolin inhibited the proliferation and invasion of breast cancer cells.

GPNMB triggers the self-renewal of spheroids to increase cell survival and the tumor-forming ability of macrophages interacting with tumor cells, and GPNMB activates the expression of the IL-33cytokine and its receptor, IL-1R1L, through CD47 and is sufficient to induce tumor spheroid formation [34]. In addition, GPNMB is a prognostic indicator and is assessed to identify recurrence in triple-negative breast cancers [35]. A previous study also found that the HDAC inhibitor, trichostatin A, induced apoptosis by inhibiting GPNMB expression in gastric cancer [36]. Dihydrolipoamide S-succinyltransferase (DLST) is the E2 transferase of the α-ketoglutarate (α-KG) dehydrogenase complex. Silencing DLST induces apoptosis and decreases cell growth in T-cell acute lymphoblastic leukemia [37]. It has been demonstrated that luteolin induces apoptosis and autophagy by regulating the p38, JNK and Akt pathways in macrophage-related diseases [38]. In addition, luteolin mediates apoptosis through the modulation of histone H3 acetylation activity in leukemia cells [39].

In KEGG pathway analysis, the present study found that luteolin-mediated DEGs were involved in T-cell leukemia virus 1 infection and differentiation. A previous study demonstrated that luteolin mediated the Th1/Th2 imbalance through the TLR4/NF-κB signaling pathway. Furthermore, luteolin inhibited the expression of Th2 transcription factor GATA-3 to inhibit the differentiation of T cells into a Th2 subset. Therefore, it is suggested that luteolin plays a crucial role in the differentiation of T cells [40]. Luteolin has also been shown to significantly activate the expressions of cyclooxygenase 2, phosphorylated STAT1 and phosphorylated STAT3 and to promote the differentiation of Th cells into Th17 cells [41]. Therefore, it was considered that luteolin plays a main role in the differentiation of T cells.

## 5. Conclusions

In conclusion, the present study reported the upregulated/downregulated mRNAs regulated by luteolin in MDA-MB-231 and MCF-7 breast cancer cells. These results provide novel insight into the effects of luteolin on breast cancer and may aid in the development of novel therapeutic strategies to prevent and treat breast cancer.

## Figures and Tables

**Table 1:** Genes with significant changes in luteolin-treated MCF-7 and MDA-MB-231 cells.

Official Gene Symbol	Gene Name	MCF-7 Fold Change (Luteolin/Control)	MDA-MB-231 Fold Change (Luteolin/Control)	Gene Expression
ENST00000438070	*Homo sapiens* cDNA 5′, mRNA sequence [CB265963]	4.127925665	7.321832	up
TMEM59L	transmembrane protein 59-like	2.667702366	4.777713	up
Lnc-CHID1-1	cDNA FLJ44677 fis, clone	2.310667214	3.432382	up
VPREB3	pre-B lymphocyte 3	2.229125449	3.137973	up
Lnc-PPP1R32-1	linc|LNCipedia lincRNA	2.14699303	3.129121	up
FCGBP	Fc fragment of IgG binding protein	2.125306475	2.639865	up
A_33_P3223860	Unknown	2.088463106	2.586989	up
ENST00000426023	*Homo sapiens* cDNA [FLJ32335]	−2.037285761	−2.6294	down
A_19_P00803499	Unknown	−2.041876695	−2.14134	down
GPNMB	glycoprotein (transmembrane) nmb	−2.065156727	−2.75712	down
RAB5C	RAB5C, member RAS oncogene family	−2.088751541	−2.03856	down
COL12A1	collagen, type XII, alpha 1	−2.11607192	−2.6307	down
LINC01116	long intergenic non-protein coding RNA 1116	−2.121113679	−2.09347	down
PID1	phosphotyrosine interaction domain containing 1	−2.132012086	−2.88883	down
PPP3R1	protein phosphatase 3, regulatory subunit B, alpha	−2.145744297	−2.30164	down
OXTR	oxytocin receptor	−2.195093084	−3.77586	down
INCENP	inner centromere protein antigen 135/155 kDa	−2.263751545	−2.45544	down
SERPINA1	serpin peptidase inhibitor, clade A (alpha-1 antiproteinase, antitrypsin), member 1	−2.322611225	−2.05576	down
HLA-DPB1	major histocompatibility complex, class II, DP beta 1	−2.352085002	−2.60705	down
FAM155A	family with sequence similarity 155, member A	−2.37484673	−2.73719	down
HMOX1	heme oxygenase (decycling) 1	−2.383757494	−2.25384	down
HLA-DRA	major histocompatibility complex, class II, DR alpha	−2.388556651	−4.32678	down
APOL6	apolipoprotein L, 6	−2.486493415	−6.89045	down
LMNB1	lamin B1	−2.542284469	−5.82738	down
APH1A	APH1A gamma secretase subunit	−2.561979585	−2.86107	down
MIR503HG	MIR503 host gene (non-protein-coding)	−2.673050357	−3.10265	down
SNORA79	small nucleolar RNA, H/ACA box 79	−2.6991916	−2.00797	down
ARHGEF9	Cdc42 guanine nucleotide exchange factor (GEF) 9	−2.706861919	−2.17995	down
UBE2E1	Ubiquitin-conjugating enzyme E2E 1	−2.715830339	−6.74322	down
SNORA12	*Homo sapiens* cDNA 5′ end, mRNA sequence [AA378382]	−2.960722177	−2.14457	down
ERF	Ets2 repressor factor	−3.038398994	−2.22017	down
APP	amyloid beta (A4) precursor protein	−3.405819858	−4.2484	down
SLC39A7	solute carrier family 39 (zinc transporter), member 7	−3.491704458	−3.31685	down
DLST	dihydrolipoamide S-succinyltransferase (E2 component of 2-oxo-glutarate complex)	−3.719897742	−2.18842	down
CCDC71	Coiled-coil domain containing 71	−3.727339467	−5.58472	down
AP2B1	adaptor-related protein complex 2, beta 1 subunit	−3.866732244	−2.69467	down
ATF6B	activating transcription factor 6 beta	−4.607165158	−3.32893	down
RNF121	ring finger protein 121	−4.680226478	−2.12748	down
A_33_P3241184	Unknown	−5.616014032	−5.53105	down
AKT2	v-akt murine thymoma viral oncogene homolog 2	−5.756814436	−3.69298	down
TAF15	TAF15 RNA polymerase II, TATA box binding protein (TBP)-associated factor	−7.381370191	−4.74989	down

**Table 2:** Top three networks associated with genes targeted by luteolin in breast cancer.

Network	Predominant Diseases and Functions	Number of Genes	Molecules in Network
GO: MF (Molecular Function)	peptide binding, serine-type endopeptidase inhibitor activity, clathrin adaptor activity, cAMP response element binding, mechanosensitive ion channel activity, phospholipase binding, heparin binding, zinc ion transmembrane transporter activity, S-acyltransferase activity	13	AP2B1/HLA-DRA/PPP3R1/HLA-DPB1/OXTR/SERPINA1/APP/ATF6B/FAM155A/LMNB1/GPNMB/SLC39A7/DLST
GO: BP (Biological Process)	positive regulation of chemokine biosynthetic process, neuron death, suckling behavior, regulation of protein localization to membrane, cellular transition metal ion homeostasis, amyloid-beta formation, endoplasmic reticulum unfolded protein response, lysine metabolic process, growth plate cartilage chondrocyte morphogenesis, regulation of mitotic cell cycle phase transition	16	HMOX1/AP2B1/AKT2/APP/GPNMB/OXTR/PID1/PPP3R1/SLC39A7/APH1A/ATF6B/RNF121/DLST/COL12A1/UBE2E1/GPNMB
GO: CC (Cellular Component)	COPII-coated ER to Golgi transport vesicle, clathrin-coated endocytic vesicle membrane, endosome membrane, nuclear lamina, dihydrolipoyl dehydrogenase complex, lateral element	12	SERPINA1/HLA-DRA/HLA-DPB1/AP2B1/APH1A/RAB5C/AKT2/APP/GPNMB/LMNB1/DLST/INCENP

**Table 3:** Kyoto Encyclopedia of Genes and Genomes pathway analysis of the dysregulated genes (top 20) identified in breast cancer cells.

Description	Count	*p*-Value	Genes
Tuberculosis	5	0.00003	RAB5C/HLA-DRA/PPP3R1/HLA-DPB1/AKT2
Human T-cell leukemia virus 1 infection	5	0.00007	HLA-DRA/PPP3R1/HLA-DPB1/AKT2/ATF6B
Th1 and Th2 cell differentiation	3	0.0009	HLA-DRA/PPP3R1/HLA-DPB1
Th17 cell differentiation	3	0.0014	HLA-DRA/PPP3R1/HLA-DPB1
Toxoplasmosis	3	0.0016	HLA-DRA/HLA-DPB1/AKT2
Asthma	2	0.0019	HLA-DRA/HLA-DPB1
Allograft rejection	2	0.0028	HLA-DRA/HLA-DPB1
Graft-versus-host disease	2	0.0033	HLA-DRA/HLA-DPB1
Type I diabetes mellitus	2	0.0036	HLA-DRA/HLA-DPB1
Phagosome	3	0.0037	RAB5C/HLA-DRA/HLA-DPB1
Alzheimer’s disease	4	0.0045	APH1A/PPP3R1/AKT2/APP
Intestinal immune network for IgA production	2	0.0047	HLA-DRA/HLA-DPB1
cGMP-PKG signaling pathway	3	0.0048	PPP3R1/AKT2/ATF6B
Influenza A	3	0.0051	HLA-DRA/HLA-DPB1/AKT2
Autoimmune thyroid disease	2	0.0054	HLA-DRA/HLA-DPB1
VEGF signaling pathway	2	0.0067	PPP3R1/AKT2
Viral myocarditis	2	0.0069	HLA-DRA/HLA-DPB1
Epstein–Barr virus infection	3	0.0081	HLA-DRA/HLA-DPB1/AKT2
Inflammatory bowel disease (IBD)	2	0.0081	HLA-DRA/HLA-DPB1
Amphetamine addiction	2	0.0091	PPP3R1/ATF6B
